# The evaluation of the distance between the popliteus tendon and the lateral collateral ligament footprint and the implant in Total knee Arthroplasty using a 3-dimensional template

**DOI:** 10.1186/s12891-020-03347-6

**Published:** 2020-05-22

**Authors:** Akihito Takubo, Keinosuke Ryu, Takanori Iriuchishima, Masahiro Nagaoka, Yasuaki Tokuhashi, Shin Aizawa

**Affiliations:** 1 Department of Orthopedic Surgery, Nihon University School of Medicine, Tokyo, Japan; 2 Department of Orthopedic Surgery, Nihon University Hospital, Tokyo, Japan; 3 Department of Orthopedic Surgery, Kamimoku Spa Hospital, Minakami, Japan; 4 Department of Functional Morphology, Nihon University School of Medicine, Tokyo, Japan

**Keywords:** Popliteus tendon, LCL, Footprint, Anatomy, Knee arthroplasty

## Abstract

**Background:**

The popliteus tendon (PT) or lateral collateral ligament (LCL) stabilizes the postero-lateral aspects of the knees. When surgeons perform total knee arthroplasty (TKA), PT and LCL iatrogenic injuries are a risk because the femoral attachments are relatively close to the femoral bone resection area. The purpose of this study was to evaluate the distance between the PT or LCL footprint and the TKA implant using a 3D template system and to evaluate any significant differences according to the implant model.

**Methods:**

Eighteen non-paired formalin fixed cadaveric lower limbs were used (average age: 80.3). Whole length lower limbs were resected from the pelvis. All the surrounding soft tissue except the PT, knee ligaments and meniscus were removed from the limb. Careful dissection of the PT and LCL was performed, and the femoral footprints were detected. Each footprint periphery was marked with a 1.5 mm K-wire. Computed tomography (CT) scanning of the whole lower limb was then performed. The CT data was analyzed with a 3D template system. This simulation models for TKA were the Journey II BCS and the Persona PS. The area of each footprint, and the length between the most distal and posterior point of the lateral femoral condyle and the edge of each footprint were measured. Matching the implant model to the CT image of the femur, the shortest length between each footprint and the bone resection area were calculated.

**Results:**

PT and LCL footprint were detected in all knees. The area of the PT and LCL footprints was 38.7 ± 17.7 mm^2^ and 58.0 ± 24.6 mm^2^, respectively. The length between the most distal and posterior point of the lateral femoral condyle and the edge of the PT footprint was 10.3 ± 2.4 mm and 14.2 ± 2.8 mm, respectively. The length between most distal and most posterior point of the lateral femoral condyle and the edge of the LCL footprint was 16.3 ± 2.3 mm and 15.5 ± 3.3 mm, respectively. Under TKA simulation, the shortest length between the PT footprint and the femoral bone resection area for the Journey II BCS and the Persona PS was 4.3 ± 2.5 mm and 3.2 ± 2.9 mm, respectively. The shortest length between the LCL footprint and the femoral bone resection area for the Journey II BCS and the Persona PS was 7.2 ± 2.3 mm and 5.6 ± 2.1 mm, respectively. The PT attachment was damaged by the bone resection of the Journey II BCS and the Persona PS TKA in 3 and 9 knees, respectively.

**Conclusion:**

The PT and LCL femoral attachments existed close to the femoral bone resection area of the TKA. To prevent postero-lateral instability in TKA, careful attention is needed to avoid damage to the PT and LCL during surgical procedures.

## Background

It is commonly known that the popliteus tendon (PT) or lateral collateral ligament (LCL) stabilizes the postero-lateral aspects of the knee [1–6]. Anatomically, The PT attaches to the lateral surface of the lateral femoral condyle on the femoral side and the popliteus muscle adheres to the posterior surface of the tibial bone. The popliteus hiatus runs from the PT attachment to the posterior of the lateral condyle. Therefore, the PT doesn’t run through the popliteal hiatus when the knee is extended, the PT located in popliteus hiatus when the knee is flexed. Functionally, the PT is considered as a primary restraint to external knee rotation [1–3, 6–8]. The LCL also attaches to the lateral surface of the lateral femoral condyle, close to the PT attachment on the femoral side, and distally it attaches to the fibula head. The LCL has been described as the primary restraint to varus knee loading [8, 9].

For the surgical treatment of osteoarthritic changes in the knee joint, total knee arthroplasty (TKA) is well known for its excellent clinical results and long-term survival rates [10–14]. When surgeons perform TKA, iatrogenic injury of the PT or LCL is a risk because the femoral attachments are relatively close to the femoral bone resection area. Several reports have evaluated PT or LCL injury occuring during TKA procedures [1, 3–5, 9, 15, 16]. Although PT or LCL iatrogenic injury was reported to be a complication in the TKA procedures, only a few studies examined the probability of excising the PT during TKA with a 2-dimensional template or during clinical TKA [1, 15]. There have been no studies using a three-dimensional (3D) evaluation and no studies evaluating the distance between the femoral attachments and the femoral bone resection area.

The purpose of this study was to determine the distance between the PT or LCL footprint and the TKA implant using a 3D template system, and to evaluate any significant differences based on the implant model, of which there are several kinds at present. This information may help surgeons choose the best implant. The 3D template system is used for preoperative planning when performing TKA. The valgus angle to the femoral shaft, rotation of the femur, and flexion-extension can be set freely. If the operation is performed as planned, it is possible to ascertain exactly how the implant will be placed from a three- dimensional perspective. Clarifying this issue would help to prevent iatrogenic PT or LCL injury during TKA surgery. We selected two models for our study: the Journey II BCS (Smith and Nephew Co., Ltd.) and the Persona PS (Zimmer-Biomet Co., Ltd.). Both models are popular choices for TKA. The reason for our selection was that both implants were performed using a common osteotomy method, and the amount of bone resection is relatively minimal in Journey II BCS TKA and relatively maximal in Persona PS TKA. In standard bone resection, the distal and posterior of femoral condyle were cut to the same size as the implant. The hypothesis was that when performing TKA, the PT and LCL attachment will be damaged if the femur is cut to the size of the implant at a common installation angle.

## Methods

**Fig. 1:**
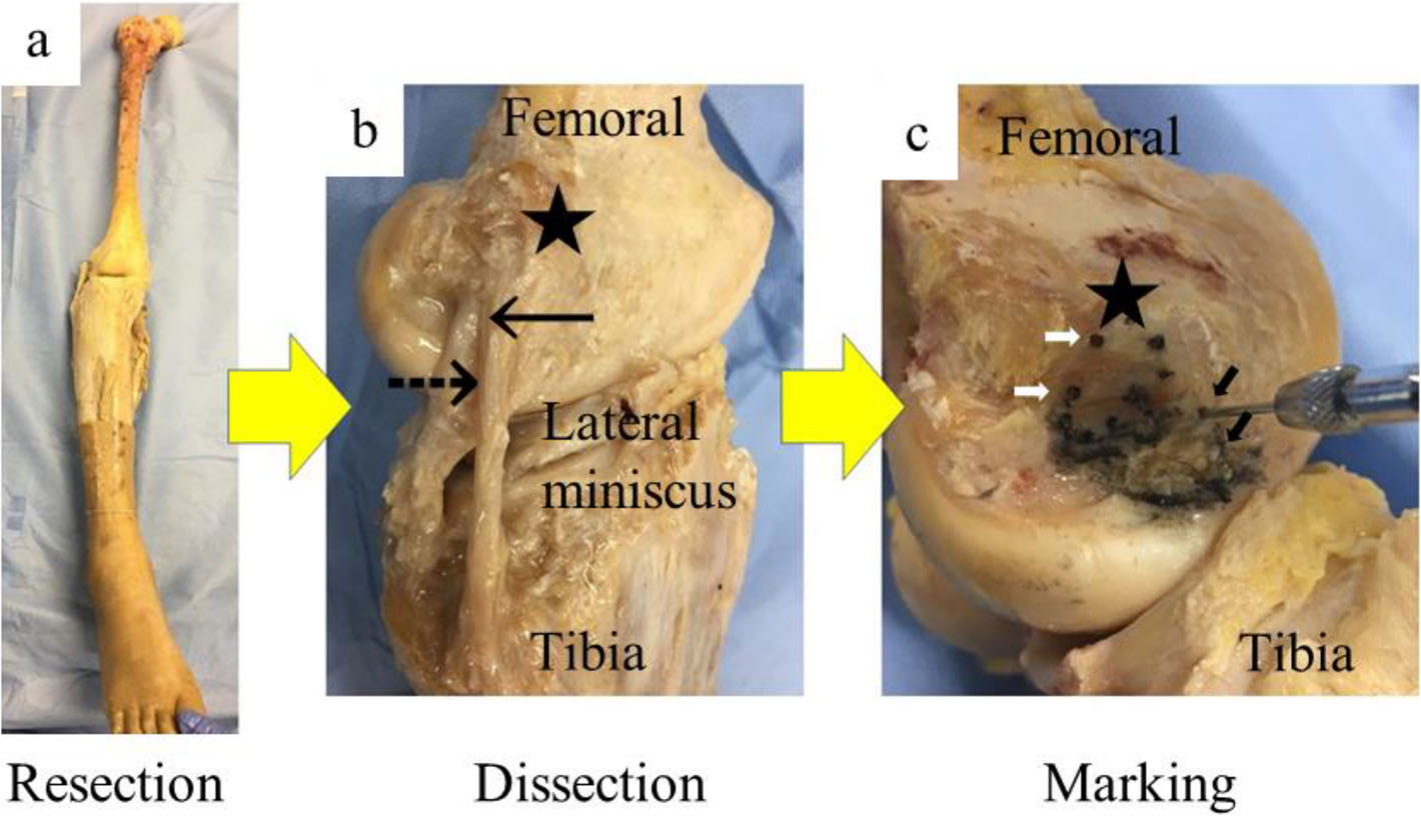
a) The right lower limb. Soft tissues without ligaments and meniscus around the knee are resected. b) lateral view of the knee. c) PT and LCL are resected. PT and LCL attachments are marked. ★; lateral epicondyle. →; LCL. ⇢; PT. ➡; Marking of the PT footprint. ⇨; Marking of the LCL footprint.

Eighteen (18) non-paired formalin fixed cadaveric lower limbs were used (9 males and 9 females). The cadavers were provided by the Department of Anatomy (Nihon University School of Medicine). While no detailed history was known, we excluded those that exhibited previous lower limbs surgery and a severe deformity during the dissection. Sixteen right lower limbs and two left lower limbs were used. Right lower limbs were preferred choice, but if the right lower limb could not be used due to previous surgery or deformation, the left lower limb was selected. The mean age at the time of death was 80.3 (range 54–90). Whole length lower limbs were resected from the pelvis. All the surrounding soft tissue except the PT, knee ligaments and meniscus were removed from the limb. Careful dissection of the PT and LCL was performed. Because the both attachments are adjacent and near the attachment, the LCL runs just outside the PT, in order to clarify each footprint first, ink was applied to the visible part of the LCL at a 2-3 mm distance from the next mark, then the LCL was cut and flipped, and ink was applied to the non-visible part. The PT attachment was similarly inked. Each inking point was periphery marked with a 1.5 mm K-wire (Fig. 1). This size was chosen because the holes smaller than 1.5 mm will not appear clearly in CT images. Both attachments were excised perfectly to pinpoint the drill hole. Computed tomography (CT) scanning of the whole lower limb was then performed (Aquilion OneTM. Toshiba Medical System, Tokyo, Japan). The CT data was analyzed with 3D template system (Zedknee software: LEXI co., Ltd. Tokyo, Japan) [17, 18]. The area of each footprint, and the length between the most distal and the most posterior point of the lateral femoral condyle and the edge of each footprint were measured. The areas were calculated using Image J software (National Institutes of Health, Bethesda, Maryland, USA). The length between the most distal and the most posterior point of the lateral femoral condyle and the edge of each footprint were measured by a coronal 2D slice and a sagittal 2D slice. The accuracy of the area measurement was less than 0.1mm^2^.

In the 3D template system, the simulation models for TKA were the Journey II BCS and the Persona PS. In the bone cut simulation bone, distal femoral bone cut thickness was 7.0 mm for the Journey II BCS and 9.0 mm for the Persona PS, respectively. Posterior bone cut thickness was 7.4 mm for the Journey II BCS and 10.0 mm for the Persona PS [11]. In the TKA simulating, the valgus angle to the femoral shaft was 6° and the external rotation angle was 3° from the posterior femoral condyle axis for both models. The implant size was such that it was not notched when the condyle was combined with the implant in the sagittal image.

**Fig. 2:**
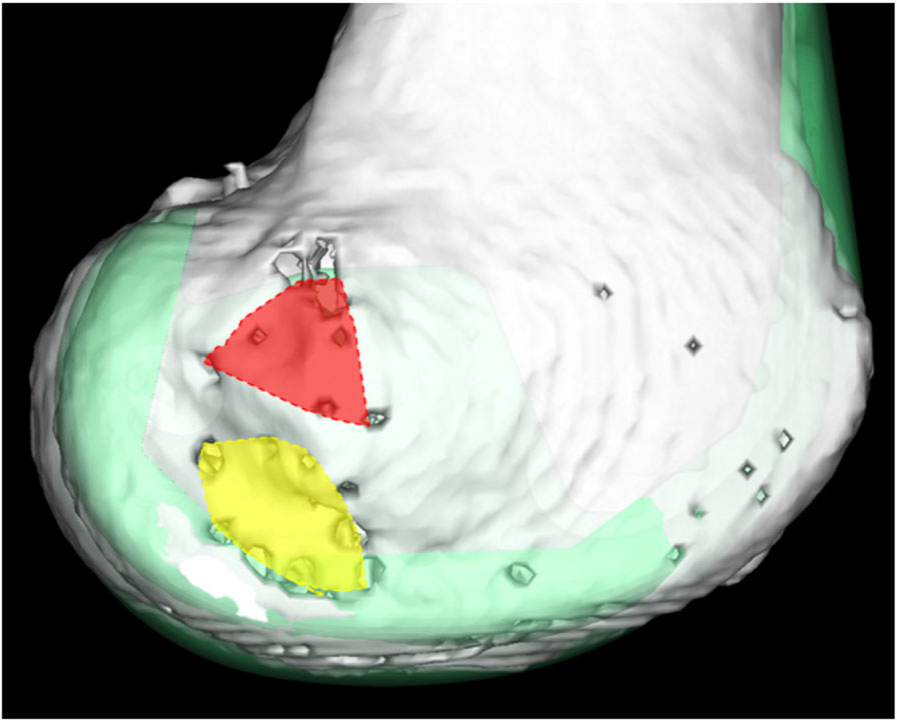
Lateral view of the femoral condyle after 3D template. Red area is the LCL footprint. Yellow area is the PT footprint. Green area is the TKA implant. PT footprint is overlapped with the TKA implant. Although there are some small holes on the anterior aspect of the lateral condyle, referring to the 2D slice only, the holes that penetrate into the femur were recognized as the attachment part

When the implant model was matched to the CT image of the femur, first the overlap between each footprint and implant was evaluated (Fig. 2). In the limbs which did not show overlap between the footprint and the both implants, the shortest length between the osteotomy and the edge of each footprint were measured by coronal 2D slice or sagittal 2D slice. We analyzed whether there was a statistically significant difference between the two models.

Those measurements were performed three times by two researchers and the average value was taken.

### Statistical analysis

Data are presented as mean ± standard deviations. Man-Whitney’s U test was performed to compare the footprint edge-implant distance between Journey II BCS and Persona PS. It was assumed that there was statistical significance when *P* < 0.05. All statistical data were calculated with SPSS 19.0 (SPSS Inc., Chicago, IL).

## Results

PT and LCL footprint were detected in all knees. The area of the PT and LCL footprint was 38.7 ± 17.7 mm^2^ (range 21.3–79.8) and 58.0 ± 24.6 mm^2^ (range 26.6–117.4), respectively. The length between the most distal and the most posterior point of the lateral femoral condyle and the closest edge of the PT footprint was 10.3 ± 2.4 mm (range 5.7–12.8) and 14.2 ± 2.8 mm (range 7.2–17.8), respectively. The length between the most distal and the most posterior point of the femur and the closest edge of the LCL footprint was 16.3 ± 2.3 mm (range 11.0–19.4) and 15.5 ± 3.3 mm (range 10.5–21.0), respectively.

In the TKA simulation, the PT footprint was overlapped with the implant in the Journey II BCS in 3 knees and in the Persona PS in 9 knees.

In knees exhibiting no overlap, the shortest length between the PT footprint and the implant was 4.3 ± 2.5 mm (range 0–6.9) for the Journey II BCS and 3.2 ± 2.9 mm (range 1.4–4.4) for the Persona PS. The shortest length between the LCL footprint and the femoral bone resection area was 7.2 ± 2.3 mm (range 3.2–11.7) for the Journey II BCS and 5.6 ± 2.1 mm (range 3.3–10.6) for the Persona PS (Table 1). In the comparison between the Journey II BCS and the Persona PS, no significant difference was seen in the lengths constituting the shortest distances from either the PT or LCL footprint edges to the implants.

**Table 1:**
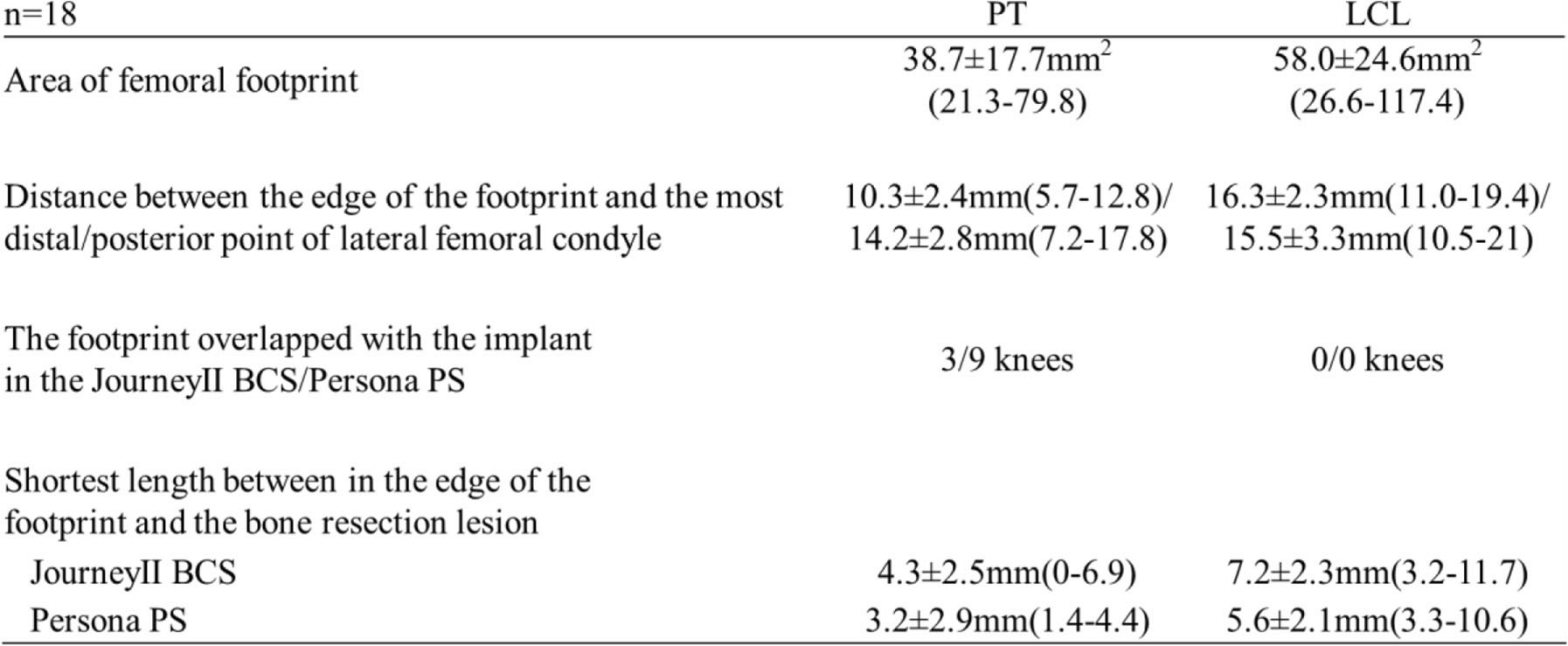
The result of the measurement

The data presented as mean ± SD (range) except the footprint overlapped with the implant.

## Discussion

The most important finding of this study was that during TKA simulation using a 3D template, approximately 17% of knees in the Journey II BCS, and 50% of knees in the Persona PS showed overlap between the PT femoral footprint and the implant. In these knees, a potential risk of iatrogenic PT footprint injury exits. To prevent postero-lateral instability in TKA, careful attention is needed to avoid PT and LCL injures in such procedures.

Historically, several studies have been conducted evaluating femoral PT or LCL footprint anatomy [6–8, 15, 16]. Takeda et al. using 3D-CT evaluation with 26 cadavers reported that the average area of PT and LCL footprint were 55.8 ± 25.0 mm^2^ and 52.5 ± 24.2 mm^2^ [8]. LaPrade et al. using computer-controlled video motion analysis captures systems with ten cadavers reported that the average area of PT and LCL footprint were 0.59cm^2^ (range 0.53–0.62cm^2^) and 0.48 cm^2^ (range 0.43–0.52cm^2^), respectively [5]. Takahashi et al. using digital camera image of the femoral lateral condyle with 21 cadavers, reported that the average area of PT footprint was 51.4 ± 12.0 mm^2^ (range 30.8–70.2 mm^2^). They reported that the average distance from the PT to the distal articular surface and the posterior articular surface was 10.2 mm ± 2.4 (range 6.5 to 16.2 mm) and 15.1 mm ± 1.9 (range 11.7 to 19.0 mm), respectively [15]. Tantavisut et al. researched gap changes after popliteus tendon resection in TKA with 14 fresh cadavers. Using a digital vernier caliper for measurement, they reported that the mean distance between the most distal femoral attachment of the PT and the most distal lateral condyle was 8.9 mm (range 6.4–10.5 mm), and that the distance from the most posterior femoral attachment of the PT to the posterior lateral femoral condyle was 11.5 mm (range 9.5–14.0 mm) [16]. In the present study, the area of the PT was smaller and the area of the LCL was similar compared with previous anatomical studies. In addition, the length between the most distal and the most posterior point of the lateral femoral condyle and the edge of the PT footprint was equivalent to that reported in Takahashi’s study, which was also conducted in a Japanese population. However, the measurements in our study tended to be larger compared with Tantavisut’s study. As the cadavers in their study were from a Thai population, ethnicity may be a likely reason for this difference, in addition to the measurement method in their study.

Although the excellent anatomical evaluation has been reported concerning PT or LCL morphology, to the best of our knowledge, not many studies have approached the correlation of morphology with TKA implants. In the Journey II BCS, distal and posterior bone cut thickness was 7.0 mm and 7.4 mm, respectively. In the Persona PS, the distal and posterior bone cut thickness was 9.0 mm and 10.0 mm, respectively. In femoral PT footprint placement, the standard thickness of bone resection in TKA presents a potential risk of iatrogenic femoral PT footprint injury. However, considering that the femoral LCL footprint is located more proximal and anterior than the PT footprint, the standard thickness of bone cut resection is potentially unlikely to result in injury to the LCL footprint. In this study, the PT footprint was overlapped by the bone resection in the Journey II BCS in 3 knees, and by the bone resection in the Persona PS in 9 knees. However, no knees exhibited overlap between the LCL footprint and TKA bone resection in either model.

Takahashi et al. evaluated the risk of excising the femoral insertion of the PT during primary TKA [15]. They colored the PT footprint, then captured a lateral image of the femur. The lateral image and the template of the femoral component were overlaid. TKA templates were Genesis II (Smith & Nephew Co., Memphis, TN, USA), Nexgen (Zimmer Co., Warsaw, IN, USA), low contact stress (LCS, DePuy Co., Warsaw, IN, USA), PFC Σ (DePuy Co., Warsaw, IN, USA), Scorpio (Stryker Co., Kalamazoo, MI, USA), and Vanguard (Biomet Co., Warsaw, IN, USA). The LCS was the only TKA template that preserved the femoral insertion of the PT. For many kinds of implants distal bone osteotomy is vertical to the femoral bone shaft, but only in LCS is the distal osteotomy line drawn at 105°towards the femoral bone shaft. In addition, the LCS has a lower component thickness than the other implants. They concluded that during primary TKA, the femoral insertion of the PT could be inadvertently excised irregardless of technical problems. The LCS design is favorable for preserving the femoral insertion of the PT. They used 2D photos taken with a digital camera from the side. However, we were able to set the mechanical axis, rotation, and flexion angle more accurately by using a 3D template system based on CT data, by which we were able to accurately measure the damage of the PT and the shortest distance from the implant to the edge of the PT. Aki et al. reported that the accidental partial and complete excision of the femoral footprint of the PT during TKA was observed in 34.2 and 17.8% of the 275 knees, respectively [1]. They used only the Nexgen template for all cases. As reported in previous studies, for many kinds of implants, osteotomy at the same thickness as the implant size is likely to damage the attachment of the PT. However, changing the type of an implant used for TKA or improving the implant may reduce the PT injury.

To our knowledge, no report has reported cases in which the bone resection line and the LCL footprint have overlapped. But Unnanuntana et al. showed that LCL injuries in TKA surgery could be the cause of knee varus instability [9]. The results of this study showed that the length between the most distal or posterior point of the lateral femoral condyle and the edge of the LCL footprint was 16.3 ± 2.3 mm and 15.5 ± 3.3 mm, respectively. Considering that the distal and posterior bone resection in the TKA is normally less than 10 mm, the risk of LCL injury may be low. However, potential risk of LCL footprint injury might exist in severe valgus knees or knees with dysplasia of the lateral femoral condyle because the distance between the distal point of the lateral condyle and the PT or LCL may be smaller.

Thus, PT injury remains a risk, but it is still unclear how PT injure will affect patients after the TKA. In clinical research, Simone et al., reported that intraoperative complete sectioning of the popliteus tendon during the performance of TKA results in decreased International Knee Society functional scores two to 3 years postoperatively [3]. Keasman et al., in clinical and cadaver combined study, reported that resectioning the PT does not appear to change the static balance of the knee. However, this study did not evaluate gaps or patients’ clinical outcomes [19]. In a cadaver study, Tantavisut et al. reported that complete PT resection in Posterior-Stabilized (PS) TKA led to an increase both flexion and extension gaps [16]. However, Ghosh et al. reported that isolated PT injury does not lead to abnormal laxity in PS TKA [4]. All of the above-mentioned studies are ones with short-term outcomes or cadaver studies, and kinematics of the knee have not been addressed. In future studies long-term results and kinematics of knees with PT injuries due to osteotomy should be evaluated.

This study has several limitations. 1) Due to the small sample size, an analyze of the precise anatomical variance was impractical. 2) Only Japanese specimens were investigated. Knee size in a Japanese population may be different from that of other populations. The size difference may affect the results because there may be a correlation between knee size and the risk of damaging the PT or LCL. 3) This study performed with CT based 3D template system, and therefore, cartilaginous tissue was not evaluated. In a clinical study, the bone cut thickness might be less than that of a CT simulation. These limitations should be considered in future studies.

## Conclusion

In conclusion, the PT and LCL femoral attachment was seen to exist close to the femoral bone resection area in TKA, and excision of the femoral PT footprint was confirmed to be a risk during standard bone resection. To prevent the postero-lateral instability in the TKA, careful attention is needed to avoid PT and LCL injuries during surgical procedures.

## Data Availability

The datasets used and/or analyzed during the current study are available from the corresponding author on reasonable request.

## Abbreviations

PT: Popliteus tendon
LCL: Lateral collateral ligament
TKA: Total knee arthroplasty
3D: Three dimensional
CT: Computed tomography
